# Spontaneous seizure and partial lethality of juvenile *Shank3*-overexpressing mice in C57BL/6 J background

**DOI:** 10.1186/s13041-018-0403-6

**Published:** 2018-10-10

**Authors:** Chunmei Jin, Yinhua Zhang, Shinhyun Kim, Yoonhee Kim, Yeunkum Lee, Kihoon Han

**Affiliations:** 1 Department of Neuroscience, College of Medicine, Korea University, 73, Inchon-ro, Seongbuk-gu, Seoul, 02841 South Korea; 2 Department of Biomedical Sciences, College of Medicine, Korea University, Seoul, 02841 South Korea

**Keywords:** Shank3 overexpression, Spontaneous seizure, Lethality, Juvenile stage

## Abstract

The SH3 and multiple ankyrin repeat domains 3 (*SHANK3*) gene encodes core scaffolds in neuronal excitatory postsynapses. *SHANK3* duplications have been identified in patients with hyperkinetic disorders and early-onset generalized tonic-clonic seizures. Consistently, *Shank3* transgenic (TG) mice, which mildly overexpress Shank3 proteins exhibit hyperkinetic behavior and spontaneous seizures. However, the seizure phenotype of *Shank3* TG mice has only been investigated in adults of the seizure-sensitive strain FVB/N. Therefore, it remains unknown if spontaneous seizures occur in *Shank3* TG mice from the early postnatal stages onward, or even in seizure-resistant strains. Clinically, generalized tonic-clonic seizures are the critical risk factor for epilepsy-associated mortality. However, the potential association between Shank3 overexpression and mortality, at least in mice, has not been investigated in detail. In the present study, we backcrossed *Shank3* TG mice in seizure-resistant C57BL/6 J strain and monitored their home-cage activities at 3 weeks of age. Of the 15 *Shank3* TG mice monitored, two exhibited spontaneous tonic-clonic seizures, and one died immediately after the seizure event. Based on this observation, we determined the survival rate of the *Shank3* TG mice from 3 to 12 weeks of age. We found that approximately 40–45% of the *Shank3* TG mice, both males and females, died before reaching 12 weeks of age. Notably, 53% and 70% of the total deaths in male and female *Shank3* TG mice, respectively, occurred in the juvenile stages. These results suggest spontaneous seizure and partial lethality of juvenile *Shank3* TG mice in seizure-resistant background, further supporting the validity of this model.

**Electronic supplementary material:**

The online version of this article (10.1186/s13041-018-0403-6) contains supplementary material, which is available to authorized users.

## Main text

Deletions, duplications, and various point mutations of the SH3 and multiple ankyrin repeat domains 3 (*SHANK3*) gene, which encodes excitatory postsynaptic core scaffolding proteins [1], are causally associated with numerous neurodevelopmental and neuropsychiatric disorders, including autism spectrum disorders (ASDs), bipolar disorder, intellectual disability, and schizophrenia [2–4]. Specifically, we previously identified two *SHANK3* duplication patients who presented with hyperkinetic disorders, such as attention deficit hyperactivity disorder (ADHD) and bipolar disorder, and early-onset generalized tonic-clonic seizures [4]. Furthermore, *Shank3* transgenic (TG) mice which mildly overexpress Shank3 proteins (by approximately 50%), exhibit mania-like hyperkinetic behavior and spontaneous seizures, recapitulating the major symptoms seen in the patients [4–6].

However, the seizure phenotype has only been investigated in adult (8 to 12-week-old) *Shank3* TG mice of FVB/N background which is a strain with high seizure sensitivity [7]. Therefore, it is unclear if spontaneous seizures occur in *Shank3* TG mice from the early postnatal stages, as in the patients, and even in other seizure-resistant strains, such as C57BL/6 J [7, 8]. Moreover, from a clinical perspective, generalized tonic-clonic seizures are the critical risk factor for epilepsy-associated mortality, such as sudden unexpected death in epilepsy (SUDEP) [9]. Although the two *SHANK3* duplication patients commonly showed generalized tonic-clonic seizures, the potential association between Shank3 overexpression and lethality, at least in *Shank3* TG mice, has not been investigated in detail.

To address these issues, we crossed *Shank3* TG mice of FVB/N strain with wild-type (WT) C57BL/6 J mice for more than ten generations. To examine behavioral seizures in the early postnatal stages, we monitored the home-cage activities of juvenile (3-week-old) *Shank3* TG mice twice per day (at 10 am and 4 pm, for one hour per each session) for a week. Of the 15 *Shank3* TG mice monitored, we found two mice exhibiting spontaneous behavioral seizures. During the seizures, both mice showed rearing, jumping, and falling with forelimb clonus (Additional file 1), which is the behavioral indication of tonic-clonic seizure (Racine’s scale 5) [10]. Notably, one of the *Shank3* TG mice died immediately after a single seizure event during our observation. None of the ten WT littermates showed any sign of behavioral seizure during a week of monitoring.


Additional file 1:Spontaneous seizure of juvenile *Shank3* transgenic mice in C57BL/6J strain. This video shows spontaneous seizure from an 3-week-old *Shank3* transgenic mouse in C57BL/6J strain. (MP4 6987 kb)


**Fig. 1:**
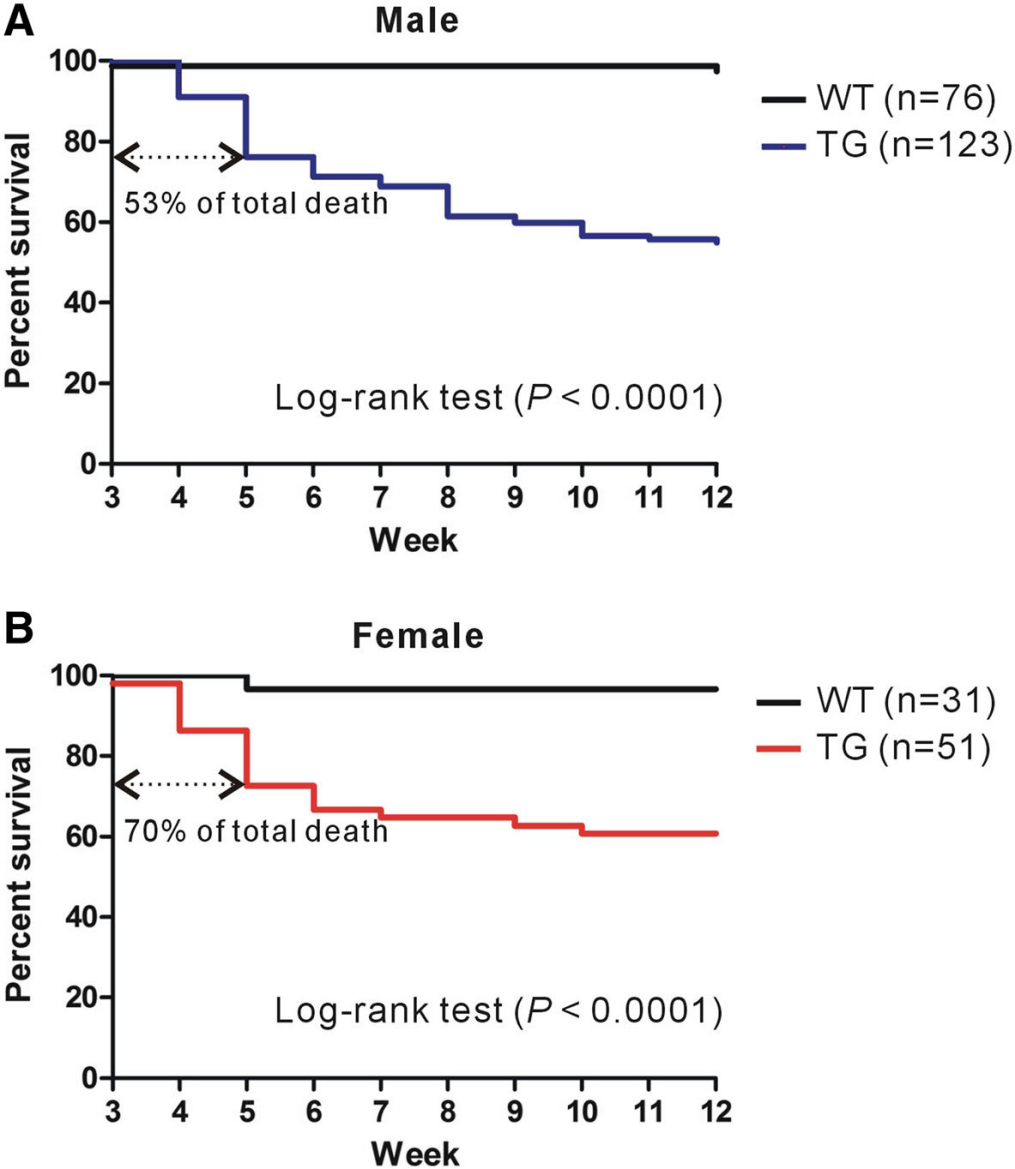
Survival plots of male and female wild-type (WT) and *Shank3* transgenic (TG) mice in C57BL/6 J background. **a** Survival plot of male WT (black line) and *Shank3* TG (blue line) mice from 3 to 12 weeks of age. Of the 123 *Shank3* TG mice, 55 died before the age of 12 weeks, and 29 (53%) died before the age of 5 weeks. Two WT mice died during counting (Log-rank test, *P* < 0.0001). **b** Survival plot of female WT (black line) and *Shank3* TG (red line) mice. Of the 51 *Shank3* TG mice, 20 died before the age of 12 weeks, and 14 (70%) died before the age of 5 weeks. One WT mouse died during counting (Log-rank test, *P* < 0.0001). Numbers for the survival plots are provided in Additional file 2

Based on our observation of the death of the *Shank3* TG mouse after spontaneous seizure, we determined the survival rates of the male and female *Shank3* TG mice, and those of their WT littermates, from postnatal 3 to 12 weeks of age. Of a total of 123 male and 51 female *Shank3* TG mice, approximately 40–45% (55 males and 20 females) died before they reached 12 weeks of age (Fig. 1a, b). Furthermore, 53% and 70% of total death in male and female *Shank3* TG mice, respectively, occurred in juvenile stages (between 3 and 5 weeks of age), which is consistent with our observation of the spontaneous seizure and subsequent death of a 3-week-old *Shank3* TG mouse. Two male (2.6% of 76) and one female (3.2% of 31) WT mice died during our counting from unknown cause.

These results suggest that *Shank3* TG mice exhibit spontaneous seizures from the early juvenile stages, and even by seizure-resistant C57BL/6 J strain, which, together with their hyperkinetic behavior, further supports the face validity [11] of these mice for modeling human *SHANK3* duplications. We did not expect that up to 40–45% of the *Shank3* TG mice would die before the age of 12 weeks. Clinically, SUDEP is the most common cause of mortality in patients with epilepsy [9]. Thus far, several genetic models of SUDEP have been established in which mostly ion channel genes are deleted or mutated [12]. If sufficiently validated, we believe that *Shank3* TG mice may provide a unique SUDEP or epilepsy-associated lethality model with an excitatory and inhibitory synaptic imbalance [4, 13], rather than ion channel dysfunction. However, further detailed investigations, including simultaneous electroencephalography (EEG) and electrocardiogram (ECG) measurements [14], are required to confirm the causal relationship between seizure and lethality in *Shank3* TG mice.

## Additional files

Additional file 2: Supplementary materials and methods, and tables. This file includes information about the mice used in this study, and tables of numbers for the survival plots. (DOCX 27 kb)

## Abbreviations

ADHD: Attention deficit hyperactivity disorder
ASDs: Autism spectrum disorders
ECG: Electrocardiogram
EEG: Electroencephalography
SHANK3: SH3 and multiple ankyrin repeat domains 3
SUDEP: Sudden unexpected death in epilepsy
TG: Transgenic
