# Supplementary material for: Spontaneous seizure and partial lethality of juvenile Shank3-overexpressing mice in C57BL/6 J background
Source: Mol Brain. 2018 Oct 10;11:57. doi: 10.1186/s13041-018-0403-6 (PMC6180595; doi:10.1186/s13041-018-0403-6)
Supplement: Supplementary file 2 — Supplementary materials and methods, and tables. This file includes information about the mice used in this study, and tables of numbers for the survival plots. (DOCX 27 kb) [file 13041_2018_403_MOESM2_ESM.docx]

**Additional File 2.**

**Spontaneous seizure and partial lethality of juvenile *Shank3*-overexpressing mice in C57BL/6J background**

Chunmei Jin, Yinhua Zhang, Shinhyun Kim, Yoonhee Kim, Yeunkum Lee and Kihoon Han

**Materials and Methods**

**Mice**

The *EGFP* (enhanced green fluorescent protein)*-Shank3* transgenic (TG) mice used in this study have been described previously [[1-4](#_ENREF_1)]. The male wild-type (WT) and *Shank3* TG mice were bred and maintained in a C57BL/6J background according to the Korea University College of Medicine Research Requirements, and all the experimental procedures were approved by the Committees on Animal Research at the Korea University College of Medicine (KOREA-2016-0096). The mice were fed and had access to water *ad libitum* and were housed under a 12-h light-dark cycle (light on at 8 am and off at 8 pm).

**References**

1. Han K, Holder JL, Jr., Schaaf CP, Lu H, Chen H, Kang H et al. SHANK3 overexpression causes manic-like behaviour with unique pharmacogenetic properties. Nature. 2013;503(7474):72-7. doi:10.1038/nature12630.

2. Lee Y, Kim SG, Lee B, Zhang Y, Kim Y, Kim S et al. Striatal Transcriptome and Interactome Analysis of Shank3-overexpressing Mice Reveals the Connectivity between Shank3 and mTORC1 Signaling. Front Mol Neurosci. 2017;10:201. doi:10.3389/fnmol.2017.00201.

3. Lee B, Zhang Y, Kim Y, Kim S, Lee Y, Han K. Age-dependent decrease of GAD65/67 mRNAs but normal densities of GABAergic interneurons in the brain regions of Shank3-overexpressing manic mouse model. Neurosci Lett. 2017;649:48-54. doi:10.1016/j.neulet.2017.04.016.

4. Lee Y, Kang H, Lee B, Zhang Y, Kim Y, Kim S et al. Integrative Analysis of Brain Region-specific Shank3 Interactomes for Understanding the Heterogeneity of Neuronal Pathophysiology Related to SHANK3 Mutations. Front Mol Neurosci. 2017;10:110. doi:10.3389/fnmol.2017.00110.

**Supplementary Tables**

Numbers for the survival plots of male and female WT and *Shank3* TG mice.

Table S1. Male

| **Weaning** | **Week** | **3** | **4** | **5** | **6** | **7** | **8** | **9** | **10** | **11** | **12** |
| --- | --- | --- | --- | --- | --- | --- | --- | --- | --- | --- | --- |
| 76 WT | Dead | 1 | 0 | 0 | 0 | 0 | 0 | 0 | 0 | 0 | 1 |
|  | Survive | 75 | 75 | 75 | 75 | 75 | 75 | 75 | 75 | 75 | 74 |
| 123 TG | Dead | 0 | 11 | 18 | 6 | 3 | 9 | 2 | 4 | 1 | 1 |
|  | Survive | 123 | 112 | 94 | 88 | 85 | 76 | 74 | 70 | 69 | 68 |

Table S2. Female

| **Weaning** | **Week** | **3** | **4** | **5** | **6** | **7** | **8** | **9** | **10** | **11** | **12** |
| --- | --- | --- | --- | --- | --- | --- | --- | --- | --- | --- | --- |
| 31 WT | Dead | 0 | 0 | 1 | 0 | 0 | 0 | 0 | 0 | 0 | 0 |
|  | Survive | 31 | 31 | 30 | 30 | 30 | 30 | 30 | 30 | 30 | 30 |
| 51 TG | Dead | 1 | 6 | 7 | 3 | 1 | 0 | 1 | 1 | 0 | 0 |
|  | Survive | 50 | 44 | 37 | 34 | 33 | 33 | 32 | 31 | 31 | 31 |
